# Prevalence rate of Metabolic Syndrome in a group of light and heavy smokers

**DOI:** 10.1186/1758-5996-5-28

**Published:** 2013-05-30

**Authors:** Hellas Cena, Antonella Tesone, Rosanna Niniano, Isa Cerveri, Carla Roggi, Giovanna Turconi

**Affiliations:** 1 Department of Public Health, Experimental and Forensic Medicine, Section of Human Nutrition, University of Pavia, Via Bassi 21, 27100, Pavia, Italy; 2 Respiratory Pathophysiology Unit, Policlinico San Matteo IRCCS, Viale Golgi, 19, 27100, Pavia, Italy

**Keywords:** Metabolic syndrome, Smoking habit, Insulin resistance, Overweight, Waist circumference

## Abstract

**Background:**

Smoking is an important cause of morbidity and mortality worldwide. It is widely accepted as a major risk factor for metabolic and cardiovascular disease. Smoking reduces insulin sensitivity or induces insulin resistance and enhances cardiovascular risk factors such as elevated plasma triglycerides, decreases high-density lipoprotein cholesterol and causes hyperglycemia. Several studies show that smoking is associated with metabolic abnormalities and increases the risk of Metabolic Syndrome. The aim of this study was to estimate the prevalence of the metabolic syndrome in a group of light and heavy smokers, wishing to give up smoking.

**Methods:**

In this cross-sectional study all the enrolled subjects voluntary joined the smoking cessation program held by the Respiratory Pathophysiology Unit of San Matteo Hospital, Pavia, Northern Italy.

All the subjects enrolled were former smokers from at least 10 years and had no cancer or psychiatric disorders, nor history of diabetes or CVD or coronary artery disease and were not on any medication.

**Results:**

The subjects smoke 32.3 ± 16.5 mean Pack Years. The prevalence of the metabolic syndrome is 52.1%: 57.3% and 44.9% for males and females respectively. Analysing the smoking habit influence on the IDF criteria for the metabolic syndrome diagnosis we found that all the variables show an increasing trend from light to heavy smokers, except for HDL cholesterol. A statistical significant correlation among Pack Years and waist circumference (R = 0.48, p < 0.0001), Systolic Blood Pressure (R = 0.18, p < 0.05), fasting plasma glucose (R = 0.19, p < 0.005) and HDL cholesterol (R = −0.26, p = 0.0005) has been observed.

**Conclusions:**

Currently smoking subjects are at high risk of developing the metabolic syndrome.

Therapeutic lifestyle changes, including smoking cessation are a desirable Public health goal and should successfully be implemented in clinical practice at any age.

## Background

Smoking is an important cause of morbidity and mortality worldwide. Currently, tobacco is the second leading cause of death in the world, accounting for about 5 million deaths annually, equivalent to 1 out of 10 adults worldwide [1].

It is widely accepted as a major risk factor for metabolic and cardiovascular disease [2]. Previous studies have shown that smoking reduces insulin sensitivity or induces insulin resistance [3,4] and enhances cardiovascular risk factors such as elevated plasma triglycerides, decreases high-density lipoprotein cholesterol (HDL-C) and causes hyperglycemia [5-8]. Furthermore, several studies show that smoking is associated with metabolic abnormalities and increases the risk of Metabolic Syndrome (MBS) [9-11]. This syndrome is associated with multiple metabolic alterations and hemodynamic disorders. Weitzman et al. [12] have demonstrated for the first time a dose-responsive, nicotine-confirmed relationship between tobacco smoke and the severity of MBS, also in adolescents, reporting that the exposure to tobacco smoke, whether by active or passive smoking, is associated with a 4-fold increase in the risk of MBS among adolescents who are overweight or at risk for overweight.

Saarni et al. [13] investigated the association of adolescent smoking with overweight and abdominal obesity in adulthood, reporting that smoking is a risk factor for abdominal obesity among both genders and for overweight in women.

In Kawada’s 1-year follow-up study [14], current smokers have a higher risk of MBS than non-smokers, independently of age, body mass index, insulin resistance, uric acid level and other lifestyle factors. In contrast, ex-smokers do not have a significantly greater risk of MBS than non-smokers.

The most effective way for smokers to decrease the risk of metabolic syndrome and cardiovascular disease is to quit smoking [15]. However, other authors highlight that smoking cessation is also associated with an increased risk of MBS due to the subsequent body weight gain [4,9].

The aim of this cross sectional study is to estimate the prevalence of the metabolic syndrome in a group of light and heavy smokers, wishing to give up smoking.

## Methods

### Sampling

In this cross sectional study, the subject recruited were all smokers attending the respiratory physio-pathological surgical outpatient clinic of the San Matteo Hospital in Pavia, Northern Italy, wishing to give up smoking. The inclusion criteria were: former smokers from at least 10 years, age range from 28 to 70 years, a medical history with no cancer or psychiatric disorders, nor history of diabetes or CVD or coronary artery disease and were not on any medication.

They were enrolled consecutively.

### Anthropometric and functional measurements

Each subject underwent a preliminary examination with a lung specialist and a subsequent examination to estimate his/her nutritional status with nutritionists: a medical doctor specialized in clinical nutrition and a registered dietitian. The following parameters were measured:

1. Body weight, measured on subjects wearing only underwear and without shoes, by means of a steel yard scale (precision ± 100 g);

2. Body height, measured on subjects without shoes by means of a stadiometer (precision ± 1 mm). BMI was calculated as the ratio between weight (in kilograms) and the square of height (in metres);

3. Four skinfold thicknesses (mid-triceps, mid-biceps, subscapular and suprailiac), measured on subjects according to standard conditions on the non-dominant body side using a Harpenden skinfold thickness calliper (resolution 2 mm); three consecutive measurements were performed and the mean of the three values was considered. The sum of the four skinfold thicknesses was computed and the body fat percentage was calculated according to the predictive equations of Durnin and Womersley [16].

4. Waist circumference, measured to the nearest mm in duplicate according to standard conditions, by placing a flexible tape midway between the lowest rib and the iliac crest. The tape was snug, but did not squeeze or compress the skin, and was parallel to the floor. The measure was collected on unclothed, relaxed subjects, after exhaling.

5. Systolic (SBP) and diastolic (DBP) blood pressure, measured according to standard conditions using a sphygmomanometer; three measurements were performed at intervals of 2–5 minutes and then mean of the three values was considered.

6. Heart rate measured according to standard conditions expressed as beats per minute (bpm), finding the pulse at the ventral aspect of the wrist on the side of the thumb (radial artery).

7. Routine haematochemical levels and any drug therapy prescribed for cigarette withdrawal by the lung specialist were recorded at every medical examination. The patient’s life style was investigated by an interview conducted by a trained dietitian, in order to evaluate the kind, frequency and intensity of physical activity.

### Smoking habits

Three smokers category have been considered: light smokers consuming till 19 Pack per Years (PY), moderate smokers ≥ 20–39 PY and heavy smokers ≥ 40 PY [17].

PY means cigarettes smoked per day times years of smoking, divided by 20 [18].

### Diagnostic criteria for metabolic syndrome

Several definitions exist for MBS [19].

In our study we used the International Diabetes Federation (IDF) clinical criteria [20], providing an universally accepted diagnostic tool that is very easy to use in clinical practice.

The IDF consensus definition includes:

▪ Ethnic specific values for waist circumference:

Central obesity is defined as waist circumference ≥94 cm for Euripides men and ≥80 cm for Euripides women, with ethnicity specific values for other groups; nevertheless if BMI is >30 kg/m^2^, central obesity can be assumed and waist circumference does not need to be measured.

▪ plus any two of the following four factors:

* raised Triglycerides (TG) level: ≥ 150 mg/dL (1.7 mmol/L), or undergoing specific treatment for this lipid abnormality;

* reduced HDL cholesterol: < 40 mg/dL (1.03 mmol/L) in males and < 50 mg/dL (1.29 mmol/L) in females, or undergoing specific treatment for this lipid abnormality;

* raised blood pressure: SBP ≥ 130 mm Hg or DBP ≥ 85 mm Hg, or undergoing a specific anti-hypertensive treatment;

* raised fasting plasma glucose (FPG) ≥ 100 mg/dL (5.6 mmol/L), or previously diagnosed type 2 diabetes (if glucose concentration is above 5.6 mmol/L or 100 mg/dL, OGTT is strongly recommended but is not necessary to define presence of the syndrome).

## Informed consent and ethical approval

Written informed consent was obtained from all participants prior to their inclusion in the study, which was performed in accordance with the ethical standards laid down in the appropriate version of the 1994 Declaration of Helsinki and approved by the University of Pavia’s Faculty of Medicine Ethical Committee.

## Statistical analysis

Comparison between males and females variables was analysed with paired Student’s *t*-test.

Pearson’s correlation coefficient was used in order to determine the association among all the variables investigated, in particular with the smoking habits (Packs per Year - PY).

Analysis of variance was used to compare light, moderate and heavy smokers.

Data were analyzed using the SPSS for PC statistical software package version 18 (SPSS Inc., Chicago, IL, USA). All the results are reported as mean ± standard deviation. The statistical significance level was set to p < 0.05 for all tests.

## Results

Of the 160 subjects assessed for eligibility, 117 (73.1% of the whole sample) were included in the study (68 males and 49 females). Forty-three individuals were excluded either because under the minimum age-range (n = 8) or affected by metabolic, cardiovascular, psychiatric and/or oncological pathologies (n = 35). The mean age of the sample was 50.1 ± 11.3 years. The mean BMI of the whole sample was 25.6 ± 4.8 kg/m^2^. Table 1 shows the age and the anthropometric characteristics of the sample.

**Table 1:** Baseline characteristics of the sample

**Variables**	**Men**	**Women**	**Total**	**p-value**
	**n = 68**	**n = 49**	**n = 117**	
Age (years)	49.7 ± 11.2	50.7 ± 11.7	50.1 ± 11.3	p = 0.639
BMI (kg/m^2^)	26.6 ± 4.9	24.3 ± 4.3	25.6 ± 4.8	p < 0.01
Fat mass (%)	25.6 ± 6.1	35.5 ± 6.7	29.7 ± 7.9	p < 0.0001
Waist circumference (cm)	97.7 ± 14.3	86.5 ± 13.7	93.0 ± 15.0	p < 0.0001
Triglycerides (mg/dL)	188 ± 125	140 ± 56	168 ± 105	p < 0.01
HDL Cholesterol (mg/dL)	43 ± 14	51 ± 12	46 ± 14	p < 0.002
Basal glycaemia (mg/dL)	100 ± 27	93 ± 18	97 ± 24	p = 0.157
SBP^1^ (mmHg)	129 ± 14	126 ± 10	128 ± 13	p = 0.250
DBP^2^ (mmHg)	83 ± 8	81 ± 6	83 ± 7	p = 0.148
Pack years (n)	35.2 ± 8.8	27.4 ± 9.6	32.3 ± 16.5	p < 0.001

^1^*SBP* Systolic Blood Pressure.

^2^*DBP* Diastolic Blood Pressure.

Waist circumference, body fat mass, BMI and PY are statistically different between gender.

Body fat percentage has been estimated by skinfolds thickness. We found that women have a higher fat mass percentage than men who show greater circumference waist and higher BMI.

A central fat distribution was found in the 73.7% of the sample; in the 71% of the same subjects the waist circumference is above the 94 and 80 cm cut off levels respectively for men and women for the European population according to IDF [21].

Biochemical and functional measurements, suggested by IDF, as MBS diagnostic criteria, are reported in Table 1.

Statistical significant differences between men and women for triglycerides and HDL cholesterol emerged. Waist circumference is positively correlated both to basal glycaemia (R = 0.41, p <0.0001), TG concentration (R = 0.26, p <0.00 5), SBP (R = 0.30, p < 0.001) and DBP (R = 0.27, p < 0.005).

The subjects smoke 32.3 ± 16.5 mean PY. In Table 2 are reported the characteristics of the sample subdivided in light, moderate and heavy smokers.

**Table 2:** Characteristics of the whole sample subdivided in light, moderate and heavy smokers.

**Variable**	**Light smokers**	**Moderate smokers**	**Heavy smokers**
	**(n = 17)**	**(n = 41)**	**(n = 59)**
Age (years)	39.2 ± 12.7*	46.2 ± 9.6	56.6 ± 7.8
BMI (kg/m^2^)	22.3 ± 3.5*	25.6 ± 4. 3	26.6 ± 5.1
Fat mass (%)	35.5 ± 10.0	40.5 ± 11.9	41.1 ± 12.5
Waist circumference (cm)	82.7 ± 12.7*	92.5 ± 13.3	96.5 ± 13.6
TG (mg/dL)	124 ± 49*	164 ± 72*	190 ± 44
HDL Col (mg/dL)	52 ± 13	46 ± 13	45 ± 15
Basal glycaemia (mg/dL)	84 ± 14	94 ± 16	104 ± 29
SBP (mmHg)	124 ± 11	126 ± 13	132 ± 13
DBP (mmHg)	82 ± 6	83 ± 8	84 ± 7
Years smoked (years)	16.6 ± 7.8*	29.1 ± 9.7*	40.9 ± 8.6
PY^3^ (n)	12.5 ± 6.2*	32.1 ± 9.9*	63.4 ± 15.0

* p < 0.05.

^1^*SBP* Systolic Blood Pressure.

^2^*DBP* Diastolic Blood Pressure.

^3^*PY* Pack Years.

Women resulted mainly light smokers (n = 13) and moderate smokers (n = 30) compared to men.

All the variables show an increasing trend from light to heavy smokers, except for HDL cholesterol, which decreases as expected. The same trend has been observed adjusting for age, gender and BMI.

In the whole sample there is a statistical significant correlation among PY and body weight (R = 0.40, p < 0.0001), BMI (R = 0.43, p < 0.0001), waist circumference (R = 0.48, p < 0.0001), fat mass (R = 0.45, p < 0.01), SBP (R = 0.18, p < 0.05), fasting plasma glucose (R = 0.19, p < 0.005).

On the other hand, an inverse correlation between PY and HDL cholesterol (R = −0.26, p = 0.0005) has been observed. HDL cholesterol is also inversely correlated to body weight (R = − 0.23,p < 0.01), BMI (R = − 0.22, p < 0.05), waist circumference (R = − 0.31, p < 0.0005), SBP (R = − 0.19,p < 0.05), fasting plasma glucose (R = − 0.12, p < 0.001) and TG (R = − 0.46, p <0.0001).

In the whole sample the MBS prevalence is 52.1%: 57.3% and 44.9% for males and females respectively.

Analysing the prevalence of the single IDF criteria for the MBS diagnosis we found that a high waist circumference value is the most frequently relieved parameter (in 88.2% of females and 91.5% of males), followed by raised TG in both genders (81.1% in females and 92.3% in males) and SBP in males (86.6%), and finally by lowered HDL levels in females (75.6%) and high DBP in males (82.4%).

Table 3 shows all the variables considered in the subjects with or without MBS. All the parameters are significantly higher (p ranges from <0.05 to < 0.0001) in patients affected by MBS, except for HDL cholesterol value, which is lower.

**Table 3:** Criteria values for Metabolic Syndrome (MBS) diagnosis, age, BMI, body fat mass, PY in the two sub samples with and without MBS (MBS vs NMBS)

**Variable**	**MBS (n 61)**	**NMBS (n 56)**	**p-value**
Age (years)	53.5 ± 10.2	46.5 ± 11.5	p = 0.0006
Males (n)	35	33	
BMI (kg/m^2^)	27.9 ± 4.4	23.1 ± 3.8	p < 0.0001
Fat mass (%)	42.3 ± 14.6	37.8 ± 8.5	p = 0.04
Waist circumference (cm)	101.0 ± 12.8	84.3 ± 12.3	p < 0.0001
Triglycerides (mg/dL)	201 ± 12.1	131 ± 66	p = 0.0002
HDL Cholesterol (mg/dL)	53 ± 15	41 ± 10	p < 0.0001
Basal glycaemia (mg/dL)	107 ± 28	86 ± 10	p < 0.0001
SBP^1^ (mmHg)	135 ± 13	121 ± 8	p < 0.0001
DBP^2^ (mmHg)	86 ± 7	79 ± 5	p < 0.0001
PY^3^ (n)	51.0 ± 28.0	36.8 ± 23.5	p < 0.05

^1^*SBP* Systolic Blood Pressure.

^2^*DBP* Diastolic Blood Pressure.

^3^*PY* Pack Years.

The sample’s life style can be described overall as sedentary, since only 42.2% of the subjects regularly walk for not more than half an hour per day and 37% of the whole sample regularly walk for not more than half an hour per week, without significant differences between gender; 80% of the sample does not practice any sports nor any programmed physical activity (78.8% of males and 81.6% of females).

## Discussion

Tobacco smoking is a major risk factor for several diseases, including MBS and consequently cardiovascular disease (CDV).

Using racial- or ethnic-specific International Diabetes Federation criteria for waist circumference, the MBS age-adjusted prevalence in the USA is 38.5% and it is higher in former smokers [22].

Currently smoking men and women are at significantly higher risk of developing MBS, increasing directly the risk of atherosclerotic cardiovascular disease development [23].

Our data further highlight the correlation of central obesity, MBS risk as well as association with obesity and smoking.

The association between smoking and MBS remains even after adjusting for other covariates, possibly a reflection of the effect of cigarette smoking on insulin resistance.

In according with Wada et al. [4] we found a positive dose–response relationship between the daily number of cigarettes and MBS prevalence rate.

This relationship is dependent on the number of cigarettes smoked daily: BMI, waist circumference, total cholesterol, TG and glucose concentration are positively associated with smoking intensity.

Cigarette smoking is an independent predictor of developing metabolic abnormalities in middle age overweight and obese adults, lowering cigarette smoking reduces risk of metabolic abnormalities, particularly in men [24]. Cigarette smoking as well as physical inactivity and obesity are associated with higher risk of the metabolic syndrome in elderly men too [25]; stopping smoking is one of the lifestyle changes, even at older ages, associated with a significant lowering risk of developing MBS [25].

In our study the prevalence of MBS in the females, although mainly light and moderate smokers compared to men, and despite significantly lower mean BMI (overall normo-weight females vs overweight males), may be partly explained by gender-difference, higher total body fat mass and fat distribution (higher waist circumferences compared to the gender specific IDF cut-points for Euripids). The women in our sample were mainly perimenopausal and it is possible that hormonal factors, in part, exert their influence on body fat distribution, as well as the age related increase of physical inactivity or higher rates of sedentary [24,26,27]. The sex difference is explained by others as a stronger anti-estrogenic effect of nicotine in women than in men [28]. Cigarette smoking, particularly smoking ≥20 cigarettes/day, has been associated with larger waist circumference and higher waist:hip ratio (WHR) in pre- and post-menopausal women after adjusting for potential confounding factors [29].

Claire C et al. [30] found that among middle-aged smokers of both sexes, waist circumference increased with number of cigarettes smoked, the authors conclude that among smokers, cigarettes smoked per day were positively associated with central fat accumulation, particularly in women [30].

Waist circumference as well as WHR is an indicator of the amount of visceral adipose tissue [31]. In our study smokers tend to have a large waist circumference that increased proportionally with the number of the pack years (R = 0.48, p < 0.0001) in agreement with Shimokata et al. [32].

Smoking seems to accelerate visceral fat accumulation and promote obesity-related disorders. Medical research has focused on visceral adiposity as a target for the management of the MBS [33]. Distribution of body fat is more important than the amount of fat as a prognostic factor for life expectancy [34].

Nicotine, carbon monoxide, and other metabolites from smoking also play important roles in insulin resistance [31]. Indeed, several studies in the past have shown that nicotine leads to insulin resistance, has an anti-estrogenic effect and increases the level of stress hormones like cortisol [35-37].

Cigarettes smoking is a strong independent risk factor for cardiovascular disease as well as for non insulin dependent diabetes mellitus [8,38].

MBS and glucose intolerance are regarded as disturbances with a common background and strong interrelations such as hyperglycemia, decreases high-density lipoprotein cholesterol (HDL-C) and elevated plasma triglycerides [39].

In according with other authors [11,40] we found that smokers had features of insulin resistance syndrome including low HDL Cholesterol, high serum triacylglicerol, high fasting glucose. In our study all these parameters are positively associated with smoking intensity: there is a statistical significant correlation among Packs Year (PY) and BMI, waist circumference, fasting plasma glucose and there is an inverse correlation between packs smoked per year and HDL cholesterol.

Low serum concentrations of high-density lipoprotein-cholesterol (HDL-C), defined as <1 mmol/L (40 mg/dL) in both sexes, or <1 mmol/L in men and <1.3 mmol/L (50 mg/dL) in women, are independent risk factors for coronary heart disease (CHD). The causes of low HDL-C include rare genetic disorders such as Tangier and secondary factors such as smoking, type 2 diabetes, metabolic syndrome and abdominal obesity [41,42].

The current International guidelines for the management of dyslipidemia recommend a change in lifestyle for people with low HDL-C, focussing on weight reduction, increased physical activity and smoking cessation [43,44], with evident benefits on the overall CVD risk and specifically on HDL-C [43].

The visceral fat accumulation and insulin resistance may represent an important link between cigarette smoking and the risk of cardiovascular disease [31]. Further research is needed in this area, but these findings indicate that more emphasis should be placed on the risk of central obesity among smokers and those who are quitting smoking. Almost any smoker is aware of the association between quitting smoking and the risk of subsequent body weight gain due to increased energy intake, decreased metabolic rate, increased physical inactivity [45] but, on the other hand not all of them know that their unhealthy lifestyle habits, such as scarce fruit and vegetable intake, excessive alcohol consumption, sedentary lead to weight gain and might partly explain why smokers tend to accumulate fat specifically in the abdominal area [31,46]. Besides a recent research reported that smoking cessation may be associated not only with increased body weight, fat mass, but also with increased lean and functional mass suggesting a novel and important finding on the benefits of quitting smoking [47].

## Limitations of current study

Our results must be interpreted in light of the study limitations. First, the study is a cross-sectional one, our results do not investigate the MBS prevalence data before and after quitting smoking. Second, inflammatory and procoagulant variable such as C-reactive protein, fibrinogen as well as citokynes concentrations were not measured.

On the other hand one of the study's strength is the use of anthropometric measurements instead of self-reported weight and height, as well as waist circumference assessment. People tend to over report their height and under report their weight, resulting in an underestimation of BMI. Under reporting of weight is more prevalent in those who are overweight or obese than in normal-weight persons [48].

## Conclusions

Currently smoking subjects are at high risk of developing the metabolic syndrome. Intervention studies offering support to smokers willing to quit through physical activity promotion and healthy diet in order to reduce smoking prevalence whereas avoid weight gain following cessation is a desirable public health goal.

Medical management and prevention programs should take into account that concerns about post cessation weight gain may deter numerous persons from quitting smoking [48], such persons should be made aware that smoking is not an efficient way to control body weight, does not help prevent obesity and could favourite visceral fat accumulation and increase the risk of metabolic syndrome.

According to our data, we suggest to specifically target, heavy smokers, because of their increased MBS risk and find support to assist them in smoking cessation. This deserves priority [49, 50] and should successfully be implemented in clinical practice at any age.

## Abbreviations

BMI: Body mass index; CDV: Cardiovascular disease; CHD: Coronary heart disease; FPG: Fasting plasma glucose; HDL-C: High-density lipoprotein cholesterol; IDF: International Diabetes Federation; MBS: Metabolic Syndrome; PY: Pack per Years; SBP: Systolic blood pressure; DBP: Diastolic blood pressure; TG: Triglycerides; WHR: Waist:hip ratio.

## Competing interests

The authors declare that they have no competing interests.

## Authors’ contributions

HC conceived the study hypothesis, supervised data analyses and wrote the manuscript, AT and RN took a lead role in the data collection. IC contributed to the study design; CR contributed to the data interpretation. GT made substantial contribution to data analysis and writing of the manuscript. All authors read and approved the final manuscript.
